# Pharmacokinetics and pharmacodynamics studies of a loading dose of cisatracurium in critically ill patients with respiratory failure

**DOI:** 10.1186/s12871-022-01571-2

**Published:** 2022-01-22

**Authors:** Panadda Panusitthikorn, Chuthamanee Suthisisang, Viratch Tangsujaritvijit, Wichit Nosoongnoen, Pitchaya Dilokpattanamongkol

**Affiliations:** 1 Department of Pharmacy, Faculty of Pharmacy, Mahidol University, 447 Sri-Ayutthaya Road, Ratchathewi, Bangkok, 10400 Thailand; 2 Acute care unit, Department of Pharmacy, Faculty of Medicine, Siriraj Hospital, Mahidol University, 2 Wanglang Road Bangkoknoi, Bangkok, 10700 Thailand; 3 Department of Pharmacology, Faculty of Pharmacy, Mahidol University, 447 Sri-Ayutthaya Road, Ratchathewi, Bangkok, 10400 Thailand; 4 Department of Critical Care Medicine, Faculty of Medicine Ramathibodi Hospital, Mahidol University, 270 Rama VI Road, Ratchathewi, Bangkok, 10400 Thailand; 5 Piyavate Hospital, 998 Rim Klong Samsen Road, Huai Khwang, Bangkok, 10310 Thailand

**Keywords:** Neuromuscular blockers, Pharmacokinetics, Critical care, Pharmacodynamics, Respiratory Failure

## Abstract

**Background:**

Previous studies reported a slow neuromuscular response with the currently recommended dose of cisatracurium in critically ill patients. Pharmacokinetic and pharmacodynamic studies of cisatracurium in critically ill patients are still limited. To our knowledge, this is the first study performed to better understand the pharmacokinetics (PKs) and pharmacodynamics (PDs) of a loading dose of cisatracurium and to identify factors that affect PK and PD changes in critically ill patients.

**Methods:**

A prospective PKs and PDs study was designed. Arterial blood samples of 10 critically ill patients with respiratory failure were collected after administering a loading dose of 0.2 mg/kg of cisatracurium. Plasma cisatracurium and laudanosine concentrations were determined using liquid chromatography-tandem mass spectrometry. The achievement of the desired pharmacodynamic response was evaluated by both 1) clinical assessment and 2) train-of-four monitoring. The PK/PD indices were analyzed for their correlation with patient’characteristics and other factors.

**Results:**

The one-compartment model best described the plasma pharmacokinetic parameters of cisatracurium. The volume of distribution at steady state and total clearance were 0.11 ± 0.04 L/kg and 2.74 ± 0.87 ml/minute/kg, respectively. The mean time to train-of-four 0/4 was 6 ± 3.86 minutes. A time to the desired pharmacodynamic response of less than 5 minutes was found in 10% of the patients. A positive correlation was found between cisatracurium concentration and albumin levels and between pharmacokinetics data and patient factors [partial pressure of carbon dioxide and respiratory alkalosis].

**Conclusion:**

The currently recommended loading dose of cisatracurium might not lead to the desired pharmacodynamic response in critically ill patients with respiratory failure.

**Trial registration:**

ClinicalTrials.gov, NCT03337373. Registered on 9 November 2017

**Supplementary Information:**

The online version contains supplementary material available at 10.1186/s12871-022-01571-2.

## Background

Neuromuscular blocking agents (NMBAs) are indicated for management in patients with respiratory failure to improve patient-ventilator synchrony and to prevent ventilator-induced lung injury. Cisatracurium, a stereoisomer of atracurium, is a nondepolarizing NMBA that is commonly used in intensive care unit (ICU) patients due to the proven efficacy from several high-quality published studies and favorable safety profiles [1–4]. Cisatracurium was used in conjunction with mechanical ventilation strategies to improve oxygenation and to reduce the inflammatory response in respiratory failure patients with acute respiratory distress syndrome (ARDS) [5, 6].

During critical illness, there are several factors, including fluid balance and multisystem organ failure, that can affect the pharmacokinetic (PK) and pharmacodynamic (PD) properties of cisatracurium. For instance, an increase in the volume of distribution resulted in inadequate tissue perfusion in septic shock patients [7]. The Hofmann elimination process was enhanced, followed by a decrease in the cisatracurium concentration at the target organ in hyperthermia and respiratory/metabolic alkalosis conditions [8, 9]. Therefore, inadequate cisatracurium concentrations in target organs may contribute to a delay in respiratory management. Since there was an inverse relationship between the potency and onset time of NMBAs and cisatracurium has a neuromuscular effect that is three times as potent as atracurium, an accurate loading dose (LD) is required to obtain a rapid onset of action [10].

For monitoring the efficacy of NMBAs in ICU patients, the clinical practice guidelines for adult critically ill patients recommend using train-of-four (TOF) combined with clinical assessment to achieve clinical goals and reduce the risk of an excessive blockade in patients on a continuous infusion of NMBAs [11]. In addition, to ensure diaphragmatic relaxation in ARDS patients, a deep neuromuscular blockade (TOF 0/4) might be required [12].

Several studies stated that cisatracurium dosing following clinical practice guideline recommendations was inadequate in critically ill patients [13–15]. PK parameters of cisatracurium in severe sepsis patients showed high interpatient variability. The volume of distribution at steady state (Vd_ss_) and clearance (CL) were 0.111 ± 0.07 L/kg and 0.31 ± 0.11 L/h/kg, respectively. The time to maximum block was 8.3 ± 2.9 min, which was slower than that of noncritically ill patients. Consequently, the author concluded that the standard dosing of cisatracurium might not have been adequate in severe sepsis patients due to a slower time to neuromuscular block [13]. However, PK and PD studies of cisatracurium in critically ill patients are still limited.

## Methods

### The aim, design and setting of the study

This study aimed to understand the PK and PD outcomes from a LD of cisatracurium in critically ill patients and to find the correlation between PK/PD and other factors. The study was a prospective and open-label study conducted in a medical ICU of an academic hospital in Thailand from December 2017 to May 2018. The protocol for the study was approved by the Committee on Human Rights Related to Research Involving Human Subjects, the Faculty of Medicine at Ramathibodi Hospital, Mahidol University. Signed written informed consent was obtained from patients or legal representatives before study recruitment.

### Participants

Intubated adult ICU patients over 18 years of age who required paralysis with cisatracurium as part of their clinical care were enrolled. The exclusion criteria included patients with a history of hypersensitivity/contraindication to any NMBA, patients with a history of neuromuscular disease, pregnant or lactating female patients, patients with an active burn injury or hypothermia defined as a tympanic body temperature lower than 36 °C, and patients receiving either an intravenous (IV) bolus of cisatracurium within 24 hours or a continuous IV infusion of cisatracurium within 48 hours before the enrollment period of the study.

### Dosing and sample collection

All patients received a 0.2 mg/kg of ideal body weight dose of cisatracurium (Nimbex^®^) as an IV LD without any repeated-dose or continuous infusion during the blood sampling period. Arterial blood samples (2 ml) were collected in an EDTA tube before and at 1, 5, 10, 12, 15, 20, 30, and 60 minutes following the administration of cisatracurium. After that, the samples were centrifuged at 3,000 g for 10 minutes at 4 °C. The plasma was removed and placed into a cryotube containing 25 μl of 2.0 mol/L of sulfuric acid and then stored at –80 °C until the analysis process began.

### Analytical methods

Cisatracurium and laudanosine plasma concentrations were determined by liquid chromatography with tandem mass spectrometry (Thermo Scientific, DIONEX UltiMate 3000 HPLC system), which was adapted from a previously described study [13]. A Luna-C_18_ (2.1 x 100 mm, 3 μm; Phenomenex, USA) with the same phase guard column (2.1 x 10 mm, 3 μm; Phenomenex, USA) was used as an analytical column. The mobile phase comprised a 0.1% formic acid aqueous solution and a 20% aqueous acetonitrile solution, and the flow rate was 0.2 ml/min. The injection volume was 15 μL. The analytical method was validated according to the Bioanalytical method validation – guidance for industry guidelines [15]. The standard curve was linear over the range of 10 to 10,000 ng/ml with precision and accuracy less than 15%. The regression coefficients (R^2^) for cisatracurium and laudanosine were 0.9955 and 0.9990, respectively.

### Pharmacokinetic analysis

Plasma concentration-time values for cisatracurium and laudanosine were analyzed simultaneously using Phoenix WinNonlin (version 8.0) software (Pharsight Corporation, Cary, North Carolina, USA). The data were explored using non-compartmental, one- and two-compartmental PK models. The principles for final model selection were a low Akaike information criterion (AIC), visual evaluation of individual cisatracurium plasma concentration-time profiles, and the precision of parameter estimations based on one sample t test or the correlation coefficient. The following PK parameters were generated using compartmental analysis: the maximal drug concentration in plasma (C_max_), CL, Vd_ss_, elimination half-life (t_1/2_), and area under the concentration-time curve (AUC). For non-steady-state data, an estimate of the Vd_ss_ was based on the last observed concentration. The elimination rate constant (k_el_) was also calculated using standard PK equations.

### Pharmacodynamics study

Before the administration of cisatracurium, sedatives were titrated to achieve the objective of the Richmond Agitation Sedation Scale -4 to -5. The desired PD response was evaluated by 1) clinical assessment and detection of ventilator synchrony judged by a physician and 2) peripheral nerve stimulation with TOF (TOF-Watch-S^®^, Organon Ltd., Ireland) monitoring. For clinical assessments, pulmonologists or intensivists were asked to identify patient-ventilator synchrony using ventilator graphics analysis and respiratory rate observations. For TOF monitoring, after cleaning the skin, electrocardiographic electrodes were placed to perform the TOF test at either the adductor pollicis or orbicularis oculi to measure twitch responses. The initial intensity of the stimulating current was set at 20 mA. The TOF test consists of four stimuli, each separated by 0.5 seconds. Detections of patient-ventilator synchrony and TOF monitoring were recorded before giving cisatracurium and at the time of blood collection. The TOF test was also performed every minute after administering cisatracurium until a TOF of 0/4 was reached.

The achievement of the desired PD response in the study was defined by both 1) physicians’ judgements for ventilator synchrony and 2) the maximum neuromuscular block from a TOF of 0/4. The time to the desired PD response after giving the LD was recorded. A post hoc analysis was performed to classify the time to the desired PD response into three groups: immediate, moderate, and delayed, which corresponded to less than 5 minutes, 5 to 10 minutes, and more than 10 minutes, respectively.

### Data collection

Patient characteristics (age, sex, weight, and Acute Physiology and Chronic Health Evaluation [APACHE] II score) and laboratory data (electrolytes, liver function tests, renal function tests, and serum albumin) on the study day were recorded. Body temperatures and arterial blood gas analysis (pH, partial pressure of carbon dioxide [pCO_2_], and partial pressure of oxygen [pO_2_] values) were collected closest to the time before cisatracurium administration. Fluid balance, calculated from differences between the total fluid intake and urine output, was measured and recorded 24 hours before enrollment. All of the collected data were evaluated as factors that affected cisatracurium plasma concentrations.

### Statistical analysis

Normally distributed data are presented as the mean and standard deviation (mean ± SD) and were compared using Student's *t test*. Nonnormally distributed data are reported as the median and interquartile range (IQR) and were analyzed using a Mann–Whitney *U test*. Spearman's rho correlation coefficient and Pearson’s bivariate correlation coefficient were tested to investigate the correlation between the cisatracurium plasma concentrations and pharmacodynamic data. All statistical data were analyzed using SPSS (version 18.0, Chicago, IL). Statistical significance was set a priori at a *p value* (95% confidence interval, CI) of less than 0.05.

## Results

### Patient population

Ten critically ill patients receiving a LD of cisatracurium as an IV bolus were enrolled. Their mean ± SD age, weight, and height were 57 ± 18 years, 55.7 ± 11.1 kg, and 164.8 ± 8.1 cm, respectively. The mean ± SD APACHE II score was 25 ± 8, with the male sex being predominant. Hypoalbuminemia was found in all of the recruited patients and ranged from 1.46 to 2.47 g/dL (mean ± SD, 1.93 ± 0.30 g/dL). All of the patients had sepsis, and half of them had septic shock. Only three patients received vasopressor therapy (< 0.5 mcg/kg/hour) during the study period. The indication for cisatracurium in this study was mainly ARDS. The median daily positive fluid balance one day before enrollment was 1170.00 ml (IQR 6.25 to 1578.25). The demographic data and clinical characteristics of the patients are shown in Table 1. Laboratory data, body temperatures, and arterial blood gas tests are shown as the mean ± SD represented for all patients and individually for each patient in Table 2, Table S1 (additional file 1), and Table S2 (additional file 2), respectively.

**Table 1:** Demographic and laboratory data and clinical diagnoses of the patients (*N*=10)

No.	Sex	Age (years)	Weight (kg)	BMI (kg/m^2^)	APACHE II	Scr (mg/dL)	Albumin (g/dL)	Cisatracurium dose (mg)	Indication for cisatracurium	Diagnosis
1	M	51	60	22.58	16	2.55	2	12	ARDS	Bacterial pneumonia
2	M	50	70.5	23.02	19	1.74	1.94	14	ARDS	Bacterial pneumonia
3	M	77	50	19.53	34	3.78	2.47	10	Facilitate procedure	Bacterial pneumonia, AKI
4	M	43	70	22.86	28	2.54	1.56	14	PEEP titration	Lymphoma, Tumor lysis syndrome, Enterocolitis
5	M	81	61.5	22.59	35	1.78	1.91	12	ARDS	Bacterial pneumonia
6	F	35	34.4	13.78	34	0.32	1.51	7	ARDS	PCP, pulmonary TB, Bacterial pneumonia, TB colitis
7	F	35	45.5	20.22	16	0.48	2.01	10	ARDS	Acute pancreatitis, Alcoholic hepatitis, AKI
8	M	67	60	22.58	24	2.91	2.43	12	ARDS	Bacterial pneumonia, ESRD
9	M	83	50	18.37	26	1.32	2.04	10	ARDS	AGE, Paralytic ileus, AKI
10	M	49	55	18.17	18	2.14	1.46	11	ARDS	Acute compartment syndrome, AKI
Mean ± SD		57 ± 18	55.7 ± 11.1	20.37 ± 3	25 ± 8	49.09 ± 41.60	1.93 ± 0.30	11.20 ± 2.10		

*AGE* Acute gastroenteritis, *AKI* Acute kidney injury, *APACHE* Acute Physiology and Chronic Health Evaluation, *ARDS* Acute respiratory distress syndrome, *BMI* Body mass index, *ESRD* End-stage renal disease, *F* Female, *M* Male, *PCP* Pneumocystis pneumonia, *PEEP* Positive end-expiratory pressure, *Scr* Serum creatinine, *TB* Tuberculosis

**Table 2:** Laboratory data, body temperature, and arterial blood gas tests of the patients (*N*=10)

Variable	Mean ± SD
AST (U/L)	89.90 ± 61
ALT (U/L)	40.50 ± 43.8
TB (mg/dl)	3.84 ± 2.9
DB (mg/dl)	2.95 ± 2.4
GGT (U/L)	168 ± 160.4
Ca (mEq/L)	7.85 ± 0.59
Mg (mEq/L)	2.06 ± 0.27
Body temperature (°C)	37.09 ± 0.69
pH	7.39 ± 0.06
pO_2_ (mmHg)	106.35 ± 78.24
pCO_2_ (mmHg)	33.36 ± 7.17
HCO_3_ (mEq/L)	16.98 ± 3.51

^a^Data were collected closest to the time before cisatracurium administration

*AST* Alanine aminotransferase, *ALT* Aspartate aminotransferase, *TB* Total bilirubin, *DB* Direct bilirubin, *GGT* Gamma-glutamyl transferase, *Mg* Serum magnesium, *Ca* Serum calcium, *HCO*_*3*_ Bicarbonate, *SD* Standard deviation

### Pharmacokinetic analysis

**Fig. 1:**
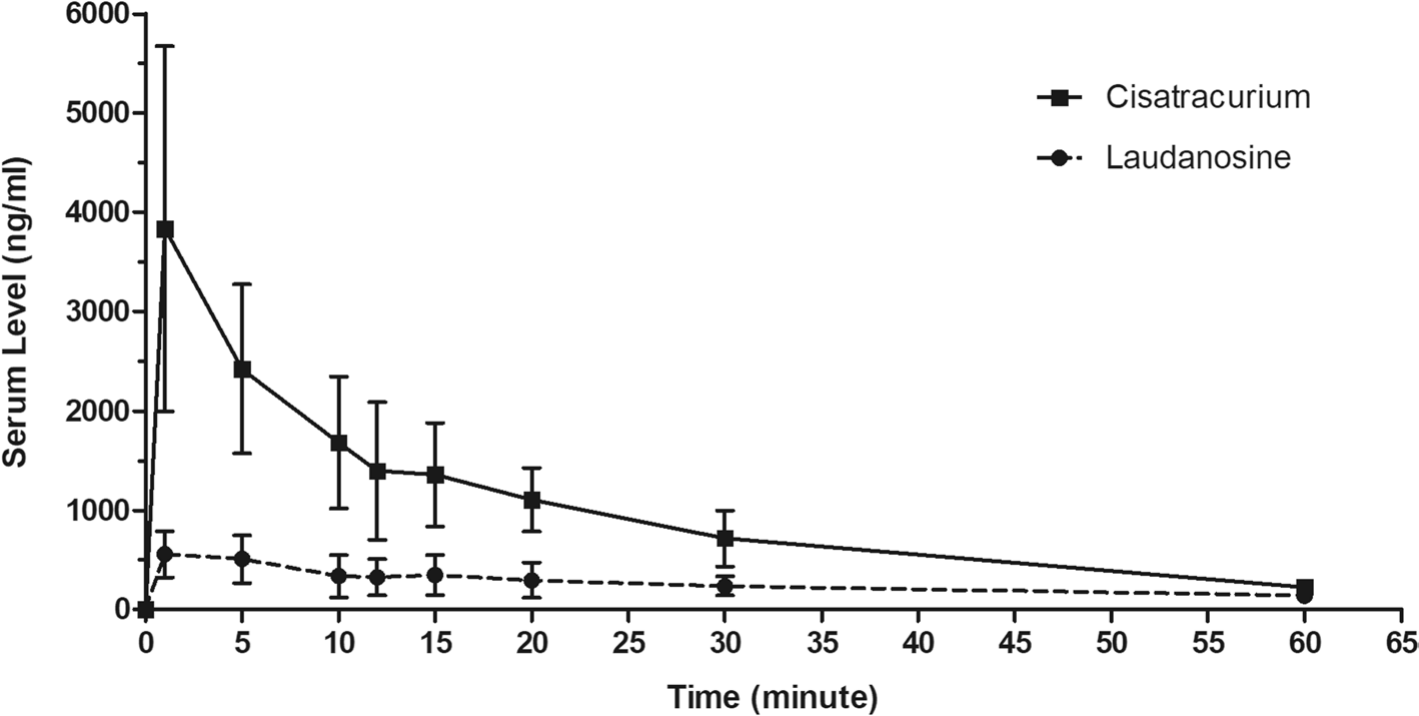
Mean and standard deviation (error bar) of the total cisatracurium and laudanosine plasma concentrations 60 minutes after an intravenous bolus of 0.2 mg/kg of cisatracurium

A one-compartment PK model fit best to explain the time course of total plasma cisatracurium concentration for the ten critically ill patients in the study. The mean ± SD of the plasma cisatracurium and laudanosine concentrations (ng/ml) versus the time profile after the IV bolus administration of 0.2 mg/kg of cisatracurium are shown in Figure 1. The individual PK parameters estimated from the PK model are summarized in Table 3. The mean concentration of laudanosine in the plasma was 626.03 ± 236.24 ng/ml.

**Table 3:** Pharmacokinetic parameters and pharmacodynamic responses to 0.2 mg/kg of cisatracurium (*N*=10)

Subject No.	Pharmacokinetic parameters^**a**^							Pharmacodynamic response			
								Clinical assessment	TOF monitoring		
	C_max_ (ng/ml)	Vd_ss_ (L)	Vd_ss_ (L/kg)	CL (ml/min)	k_el_ (min^-1^)	t_1/2_ (min)	AUC_all_ (ng·h/ml)	Groups of time to patient-ventilator synchrony^b^	Groups of time to a TOF of 0/4^b^	Time to a TOF of 0/4 (min)	TOF site
1	2115.50	5.67	0.09	162.14	0.03	24.25	1233.50	moderate	immediate	2	FN
2	3204.63	4.37	0.06	227.16	0.05	13.33	1027.19	delayed	immediate	1	UN
3	1339.09	7.47	0.15	177.40	0.02	29.18	939.48	moderate	moderate	5	UN
4	1321.15	10.60	0.15	290.34	0.03	25.30	803.67	immediate	moderate	6	UN
5	3028.18	3.96	0.06	68.60	0.02	40.04	2915.56	moderate	moderate	7	UN
6	2613.11	2.68	0.08	92.49	0.03	20.07	1261.34	moderate	delayed	13	UN
7	1399.61	7.14	0.16	83.40	0.01	59.38	1998.44	moderate	delayed	12	UN
8	1729.48	6.94	0.12	139.86	0.02	34.39	1429.99	immediate	immediate	4	UN
9	1344.94	7.44	0.15	164.29	0.02	31.37	1014.45	moderate	moderate	5	FN
10	3204.06	3.43	0.06	139.22	0.04	17.09	1316.82	moderate	moderate	5	UN
Mean ± SD	2129.97 ± 812.32	5.97 ± 2.41	0.11 ± 0.04	154.49 ± 67.61	0.03 ± 0.01	29.44 ± 13.25	1394.04 ± 629.36				

^a^Pharmacokinetic parameter estimation by one-compartmental analysis

^b^Patients were classified by time to patient-ventilator synchrony and a TOF 0/4 into the following 3 groups: 1) immediate, less than 5 minutes; 2) moderate, 5 to 10 minutes; and 3) delayed, more than 10 minutes

*AUC*_*all*_ Area under the curve from the time of dosing to the time of the last observation, *C*_*max*_ The maximal drug concentration in plasma, *CL* Clearance, *k*_*el*_ Elimination rate constant, *t*_*1/2*_ Elimination half-life, *Vd*_*ss*_ Volume of distribution at steady state, *TOF* Train-of-four, *FN* Facial nerve, *UN* Ulnar nerve

### Pharmacodynamics analysis

The PD response, evaluated by clinical assessment and TOF monitoring, and the TOF site of each patient are shown in Table 3. The mean time to a TOF of 0/4 was 6 ± 3.86 minutes. Regarding the desired PD response, 1 of the 10 patients (10%) had a time to the desired PD response of less than 5 minutes, and other patients (90%) had a time to the desired PD response of more than 5 minutes.

### Correlation of the patients’ baseline characteristics and pharmacokinetic parameters

There was no correlation between the PD data and either cisatracurium concentration or PK parameters of cisatracurium. No significant correlation was found between the PK parameters of cisatracurium and the patients’ characteristics or clinical laboratory data. The cisatracurium concentration at 1 and 30 minutes after giving cisatracurium and the albumin level had a negative correlation. The albumin level had a negative correlation with k_el_. Considering patient factors, it was observed that pCO_2_ had a statistically significant negative correlation with k_el_ and a positive correlation with t_1/2_. In addition, respiratory alkalosis had a statistically significant positive correlation with k_el_, as shown in Table 4.

**Table 4:** Correlations between the pharmacokinetic data and patient factors

Patient factor		Cisatracurium plasma concentration		PK parameters of cisatracurium	
		M1	M30	k_**el**_	t_**1/2**_
Albumin level	Correlation	-0.745	-0.683	-0.646	0.527
	*p value* (95%CI)	0.013* (-0.935 to -0.218)	0.042* (-0.917 to -0.094)	0.044* (-0.906 to -0.028)	0.177 (-0.153 to 0.868)
pCO_2_	Correlation	-0.498	-0.192	-0.699	0.778
	*p* -value (95%CI)	0.143 (-0.498 to 0.191)	0.620 (-0.733 to 0.497)	0.024* (-0.922 to -0.124)	0.008* (0.291 to 0.944)
Respiratory alkalosis	Correlation	0.485	0.053	0.636	-0.672
	*p* -value (95%CI)	0.155 (-0.152 to 0.890)	0.892 (-0.554 to 0.830)	0.048* (0.044 to 0.909)	0.033* (-0.931 to -0.188)

*statistically significant difference *p* value < 0.05

k_el_, elimination rate constant; M1, concentration of cisatracurium 1 minute after cisatracurium administration; M30, cisatracurium plasma concentration 30 minutes after cisatracurium administration; t_1/2_, elimination half-life; pCO_2_, partial pressure of carbon dioxide

## Discussion

This study was undertaken to investigate the PK parameters and PD responses of cisatracurium in critically ill patients. There were alterations in the cisatracurium PK profiles, such as a decreased volume of distribution at steady state and clearance and a comparable PD response, such as a delayed time to adequate neuromuscular response, in comparison with previous data [7]. Moreover, there were factors that affected the PK parameters, such as the albumin level, the pCO_2_, and respiratory alkalosis, which have never been mentioned in any studies.

The best-fit model to explain the generated data from our ten critically ill patients was a one-compartment model with first-order pharmacokinetics. Judgment of the appropriateness of a particular compartment model in explaining the data was warranted based on the AIC, one sample t test, correlation coefficient and visual evaluation of the fitting curve. There was a statistically significant difference in the mean AIC and concentration-time profile between the one-compartment and the two-compartment models (134.02 vs. 139.5, *p* value <0.001). Moreover, our data did not completely fit the two-compartment model. Additionally, there was no statistically significant difference between the differences in the measured concentration and predicted concentration from the one-compartment and the two-compartment models (range from -8.05 to 15.11, *p* value >0.05). Data from the concentration-time profiles of three patients (Nos. 7, 8, and 9) could not be analyzed using the two-compartment model because fit model failures were reported. Several studies showed that the two-compartment model with elimination from the central and peripheral compartments accurately described the PK parameters of cisatracurium in various populations [16, 17]. This links to the fact that Hofmann elimination is the main pathway of degradation, and the drug is also cleared by organ-dependent mechanisms [18]. There was evidence of both the one- and two-compartment models described in critically ill patients [7, 13]. However, the one-compartment PK model from this study might be explained by the lower Vd_ss_ compared with previous studies, as shown in Table 5; therefore, penetration to the peripheral compartment might be rarely observed within a short period of time.

**Table 5:** Comparison of doses, patient characteristics, and pharmacokinetic parameters of cisatracurium in critically ill patients between studies

	Our study	Liu et al.^13^	Boyd et al.^7^
Cisatracurium dose	0.2 mg/kg	0.1 mg/kg	0.1 mg/kg of an IV bolus (if necessary) and an infusion of 0.18 mg/kg/h
Characteristics of the patients	Critically ill patients with respiratory failure or ARDS (*n*=10)	Critically ill patients with severe sepsis (*n*=8)	Critically ill patients receiving mechanical ventilation (*n*=6)
Severity score: APACHE II (mean ±SD)	25 ± 8	11.9 ± 3.7	15 ± 6.7
Model	1-compartment model	1-compartment model	2-compartment model with elimination from the central compartment
PK parameters	-	-	-
**-** Vd_ss_	- 0.11 ± 0.04 L/kg	- 0.111 ± 0.07 L/kg	- 0.32 ± 0.15 L/kg
**-** CL	- 2.74 ± 0.87 ml/min/kg (0.16 ± 0.05 L/h/kg)	- 5.23 ± 1.8 ml/min/kg (0.31 ± 0.11 L/h/kg)	- 7.9 ± 2.7 ml/min/kg (0.31 ± 0.11 L/h/kg)

*APACHE* Acute Physiology and Chronic Health Evaluation, *ARDS* Acute respiratory distress syndrome, *CL* Clearance, *Vd*_*ss*_ Volume of distribution at steady state

This study confirmed the interindividual variability of cisatracurium concentrations and PK parameters in ICU patients. Cisatracurium plasma concentrations in our studied patients were higher than the concentrations, which appeared in other studies due to a maximum recommended LD of 0.2 mg/kg [7, 13]. The t_1/2_ in our study was consistent with what has been reported for other populations, which might result from a slight change in pH (7.39 ± 0.06) and controlled body temperatures in the enrollment process (37.09 ± 0.69 °C). Insignificant changes in pH and temperature resulted in a fixed k_el_, so the CL of cisatracurium is dependent on the Vd_ss_ [18]. The results showed that the Vd_ss_ was lower than the Vd_ss_ from previous studies, as presented in Table 5. This finding might be described by differences in patient characteristics between studies. There were different numbers of ARDS patients: none, one patient and eight patients (0, 17%, 80%) in the studies by Liu *et al*., Boyd *et al*., and our study, respectively. Conservative fluid management strategies were encouraged as the best practice in ARDS patients because large-volume fluid can worsen alveolar flooding and decrease gas exchange [19]. Thus, a smaller Vd_ss_ due to a lower volume of fluid resuscitation in the studied patients might occur.

There was a delay in the time to achieve the desired PD response after administering 0.2 mg/kg of cisatracurium. By TOF monitoring and the same given dose, the average time to a TOF of 0/4 in the patients was more prolonged (6± 3.86 minutes) than that of the healthy volunteers (2.7 to 3.2 minutes) [20, 21]. Similar to Liu *et al*., the average time to maximum block, defined by a percentage of the height of T1 and the T4:T1 ratio, was also prolonged (8.3 ± 2.9 minutes) in eight severe sepsis patients receiving a lower recommended dose (0.1 mg/kg). The desired PD response, assessed by the detection of ventilator synchrony and a TOF of 0/4, occurred in less than 5 minutes in only 10% (1/10 patients) of the studied patients. These findings supported that the LD of cisatracurium (0.2 mg/kg) from the current recommended guideline might be inadequate to achieve a rapid time to the desired neuromuscular response in critically ill patients with respiratory failure.

The explanation for the delayed PD response was respiratory alkalosis, which was concordant with the study of Platt *et al* [8]. There was a correlation between the pCO_2_ and k_el_, the pCO_2_ and t_1/2_ but not between the pH and t_1/2_. These might result from an insignificant change in pH from baseline in our study (from a baseline pH of 7.39± 0.06 to a pH of 7.34 ± 0.1), in contrast with a major change in pH in the Platt *et al* study (from a baseline pH of 7.35 ±0.05 to a pH of 7.5 ± 0.04). The pCO_2_ was reported to reflect the intracellular site of action of pH changes [22]. Since the rate of Hofmann elimination was increased by base molecules [23], respiratory alkalosis might be a factor that caused higher elimination at the target organ (diaphragm). For other reasons, decreasing pCO_2_ may produce a lower affinity of NMBAs to acetylcholine receptors. Furthermore, muscle relaxation might be reduced [24].

Apart from this, all of the patients had hypoalbuminemia (albumin level of 1.93 ± 0.30 g/dL). A correlation between the patients’ albumin levels and either the cisatracurium concentration or k_el_ was found in the study. Due to a reduction in the patients’ albumin levels, an increase in free drug concentrations could consequently be found [25]. Therefore, we found a higher cisatracurium level and k_el_ in hypoalbuminemia patients. Moreover, the enhanced elimination rate of free cisatracurium can cause an increase in laudanosine, a toxic metabolite that is produced. However, the data showed a higher mean C_max_ of laudanosine (626.03 ± 236.24 ng/ml after an IV bolus of 0.2 mg/kg of cisatracurium) compared to noncritically ill patients [16, 17, 26]. Additionally, the C_max_ of laudanosine in the findings was lower than the data from Boyd *et al*., who used a continuous infusion regimen for a total maximum dose of 612 mg per course of treatment (a mean of 210-1300 ng/ml). Recent studies in ARDS patients used an initial bolus dose close to 0.2 mg/kg or more without reported neurotoxic effects associated with higher laudanosine concentrations. Consequently, 0.2 mg/kg of cisatracurium has proven to be harmless for ICU patients.

This is the first PK and PD study of cisatracurium in ICU patients with respiratory failure and a high severity of illness. It is also the first study to report the desired PD response of cisatracurium by evaluating both 1) clinical assessments and physicians’ judgements for ventilator synchrony and 2) TOF monitoring. This study also provides further evidence that pCO_2_ is one of the essential factors that affects the elimination process of cisatracurium. For these reasons, a generalization of the increased LD, together with a proper neuromuscular response assessment for patient-ventilator asynchronies in real-world practice, could be applied in critically ill patients with respiratory failure.

However, there were some limitations to the study. First, the patients in this study were predominantly ARDS patients with low body weight. Other indications for NMBA therapy, including intra-abdominal hypertension in abdominal compartment syndrome, controlling muscle tone in tetanus, or targeted temperature management, could be limited for application. Second, since the clinical assessment was only recorded at a discrete time point of blood sample collection, the exact time for the neuromuscular block could not be measured. Therefore, further research could continue to explore the appropriate time to adequate neuromuscular block by continuous monitoring of patient-ventilator synchrony. Third, although ventilator graphics observations helped to recognize patient-ventilator synchrony, the expertise of the physicians was one of the major confounders in the interpretations [27]. The proper detection of patient-ventilator synchrony should be standardized, and the need for other techniques, such as esophageal pressure, to facilitate recognition of these events should be applied. In addition, the differences between TOF monitoring sites in our study, including titrating the amplitude of peripheral nerve stimulation to achieve a target TOF, might have resulted in discrepancies in PD outcomes. The same TOF monitoring site is suggested in future research. Fourth, since this study emphasizes the assessment of the efficacy of a cisatracurium LD, data regarding adverse events from cisatracurium, such as bradycardia and hypotension, which have been reported to be less than 1% [4], were not collected in the study. Other adverse events from either ventilator complications or medication should be recorded in the future. Last, no correlation was found between the PD response and either the cisatracurium concentrations or PK parameters of cisatracurium. These findings may be explained by 1) the primary objective set to find the direct PK/PD relationship and 2) the small sample size. First, the methodology in our study was initially designed to evaluate the direct PK/PD relationship. Since cisatracurium PK was described by a two-compartment model [13], the indirect link model should be considered [28]. Second, the limitation of a small amount of data might be another explanation for why the covariates were not found. Correlations would be clearly evident if the sample size and patient parameters are increased and variations in parameters are decreased. To find the correlations between the PK/PD and other factors, a larger number of patient parameters would be required to lessen variations in the parameters. Therefore, to find the appropriate dose of cisatracurium for critically ill patients, PK/PD modeling should be evaluated in future studies.

## Conclusions

A one-compartment PK model fit the concentration data best in critically ill Thai patients. In this paper, the PK parameters of cisatracurium displayed a decreased volume of distribution with reduced clearance compared with a previous study [7]. Additionally, a small percentage of patients rapidly achieved the desired PD response (monitored by clinical assessment and a TOF of 0/4). Therefore, a LD of 0.2 mg/kg of cisatracurium in critically ill respiratory failure patients might result in unsatisfactory delayed patient-ventilator synchrony, which could lead to an increase in physical distress in these patients.

## Supplementary Information

**Additional file 1: Table S1.** Laboratory data of patients (*N*=10).

**Additional file 2: Table S2.** Amplitude of the PNS data, body temperature, arterial blood gas test, type of sedation of the patients (*N*=10).

**Additional file 3: Table S3.** Total plasma concentrations of cisatracurium in 10 critically ill patients (ng/ml).

**Additional file 4: Table S4.** Total plasma concentrations of laudanosine in 10 critically ill patients (ng/ml).

**Additional file 5: Table S5** and **S6.** Correlation of patient characteristics and pharmacokinetic parameters.

## Data Availability

The datasets used and analyzed during the current study are available from the corresponding author on reasonable request.

## Abbreviations

NMBA: Neuromuscular blocking agent
ICU: Intensive care unit
ARDS: Acute respiratory distress syndrome
PK: Pharmacokinetic
PD: Pharmacodynamic
LD: Loading dose
TOF: Train-of-four
V_dss_: Volume of distribution at steady state
CL: Clearance
IV: Intravenous
AIC: Akaike information criterion
t_1/2_: Elimination half-life
AUC: Area under the concentration-time curve
K_el_: Elimination rate constant
APACHE: Acute Physiology and Chronic Health Evaluation
pCO_2_: Partial pressure of carbon dioxide
pO_2_: Partial pressure of oxygen
SD: Standard deviation
IQR: Interquartile range
