# Supplementary material for: Pharmacokinetics and pharmacodynamics studies of a loading dose of cisatracurium in critically ill patients with respiratory failure
Source: BMC Anesthesiol. 2022 Jan 22;22:32. doi: 10.1186/s12871-022-01571-2 (PMC8783433; doi:10.1186/s12871-022-01571-2)
Supplement: Supplementary file 1 — Additional file 1: Table S1. Laboratory data of patients (N=10). [file 12871_2022_1571_MOESM1_ESM.pdf]

Pharmacokinetics and pharmacodynamics studies of a loading dose of cisatracurium in critically ill patients with respiratory failure

Table S1. laboratory data of patients (N=10)

No.	AST (U/L)	ALT (U/L)	TB (mg/dl)	DB (mg/dl)	GGT (U/L)	Na (mEq/L)	K (mEq/L)	HCO_3 (mEq/L)	Ca (mEq/L)	Mg (mEq/L)
**1**	192	126	4.5	3.3	130	144	3.75	18.7	8.8	2.09
**2**	45	23	7.6	6.1	220	141	2.95	14	8.3	1.98
**3**	106	28	2.1	1.5	304	145	4.48	16	8.8	1.91
**4**	14	5	5.5	4.7	216	152	3.49	19.4	7.6	1.93
**5**	19	13	0.6	0.3	40	138	3.73	17.9	7.7	2.13
**6**	106	5	5	4.1	54	135	3.66	13.2	7.3	1.56
**7**	164	28	2.2	1.5	541	138	3.96	16.5	7.4	2.57
**8**	78	107	0.5	0.2	38	138	4.1	19.4	7.8	2.3
**9**	133	63	1.8	1	95	141	3.46	23.3	7.2	1.92
**10**	42	7	8.6	6.8	42	143	4.18	11.4	7.6	2.17
**Mean ± SD**	89.90 ± 61	40.50 ± 43.8	3.84 ± 2.9	2.95 ± 2.4	168 ± 160.4	141.5 ± 4.84	3.78 ± 0.43	16.98 ± 3.51	7.85 ± 0.59	2.06 ± 0.27

Data were collected closest to the time before cisatracurium administration

Abbreviations: AST = Alanine aminotransferase, ALT = aspartate aminotransferase, TB = total bilirubin, DB = direct bilirubin, GGT= gamma-glutamyl transferase, Na = sodium, K= serum potassium, Mg= serum magnesium, Ca = serum calcium, HCO_3 = bicarbonate, SD = standard deviation
