# Supplementary material for: Pharmacokinetics and pharmacodynamics studies of a loading dose of cisatracurium in critically ill patients with respiratory failure
Source: BMC Anesthesiol. 2022 Jan 22;22:32. doi: 10.1186/s12871-022-01571-2 (PMC8783433; doi:10.1186/s12871-022-01571-2)
Supplement: Supplementary file 2 — Additional file 2: Table S2. Amplitude of the PNS data, body temperature, arterial blood gas test, type of sedation of the patients (N=10). [file 12871_2022_1571_MOESM2_ESM.pdf]

Pharmacokinetics and pharmacodynamics studies of a loading dose of cisatracurium in critically ill patients with respiratory failure

Table S2. Amplitude of the PNS data, body temperature, arterial blood gas test, type of sedation of patients (N=10)

No.	Amplitude of the PNS (mA)*	Body temperature** (°C)	pH**	pO_2** (mmHg)	pCO_2** (mmHg)	Sedative agents
**1**	40	37.1	7.41	81	39	Propofol, Fentanyl
**2**	40	38.3	7.33	71	30	Propofol, Fentanyl
**3**	40	37.3	7.36	321.3	29.5	Midazolam
**4**	30	36.6	7.42	89.2	28.8	Fentanyl , Midazolam
**5**	25	36.4	7.35	64	40	Fentanyl , Midazolam
**6**	35	36.8	7.41	60	26	Propofol, Fentanyl
**7**	25	38.3	7.27	102.6	42.2	Fentanyl , Midazolam
**8**	35	36.9	7.4	69	40	Propofol, Fentanyl
**9**	10	36.6	7.5	77	37	Fentanyl , Midazolam
**10**	25	36.7	7.4	128.4	21.1	Fentanyl , Midazolam

*The amplitude of the PNS was titrated to reach a TOF of 4/4

** Data were collected closest to the time before cisatracurium administration

Abbreviations: mA= milliamps, pO_2 = Partial pressure of oxygen, pCO_2 = Partial pressure of carbon dioxide, PNS = Peripheral nerve stimulation
