# Supplementary material for: Pharmacokinetics and pharmacodynamics studies of a loading dose of cisatracurium in critically ill patients with respiratory failure
Source: BMC Anesthesiol. 2022 Jan 22;22:32. doi: 10.1186/s12871-022-01571-2 (PMC8783433; doi:10.1186/s12871-022-01571-2)
Supplement: Supplementary file 3 — Additional file 3: Table S3. Total plasma concentrations of cisatracurium in 10 critically ill patients (ng/ml). [file 12871_2022_1571_MOESM3_ESM.pdf]

Pharmacokinetics and pharmacodynamics studies of a loading dose of cisatracurium in critically ill patients with respiratory failure

Table S3. Total plasma concentrations of cisatracurium in 10 critically ill patients (ng/ml)

Subject	Time (minute)							
	**1**	**5**	**10**	**12**	**15**	**20**	**30**	**60**
**1**	3114.2	3598.2	2391.6	825.2	711.2	858.2	572.5	-
**2**	6639.8	2522.7	1717.8	1471.4	1400.4	1104	-	-
**3**	2541.2	1377.3	895	885.1	1209.6	815.8	536.8	209.3
**4**	2834.5	1182.7	938.9	668.1	731.9	975.6	579.6	301.8
**5**	5228.4	3358.1	2866.8	2976	2499.7	1783.1	1304	-
**6**	4613.2	3324.8	1922	1496.4	1371.8	1057.9	818.7	-
**7**	2110.4	1662.5	1591.2	1460	1532.3	940.4	544.4	-
**8**	2521.2	2681.2	863.1	1678.4	1450.6	1447.5	718.8	168.3
**9**	1961.2	2020.6	1593.2	725.2	976.1	785	393.1	-
**10**	6771	2481.2	2067.3	1782.8	1730.9	1306.6	1005.5	-
**Mean ± SD**	3521.70 ± 1646.58	2409.62 ± 902.03	1681.01 ± 704.59	1388.58 ± 733.66	1357.12 ± 554.51	1107.79 ± 337.4	719.27 ± 283.95	226.5 ± 68.39

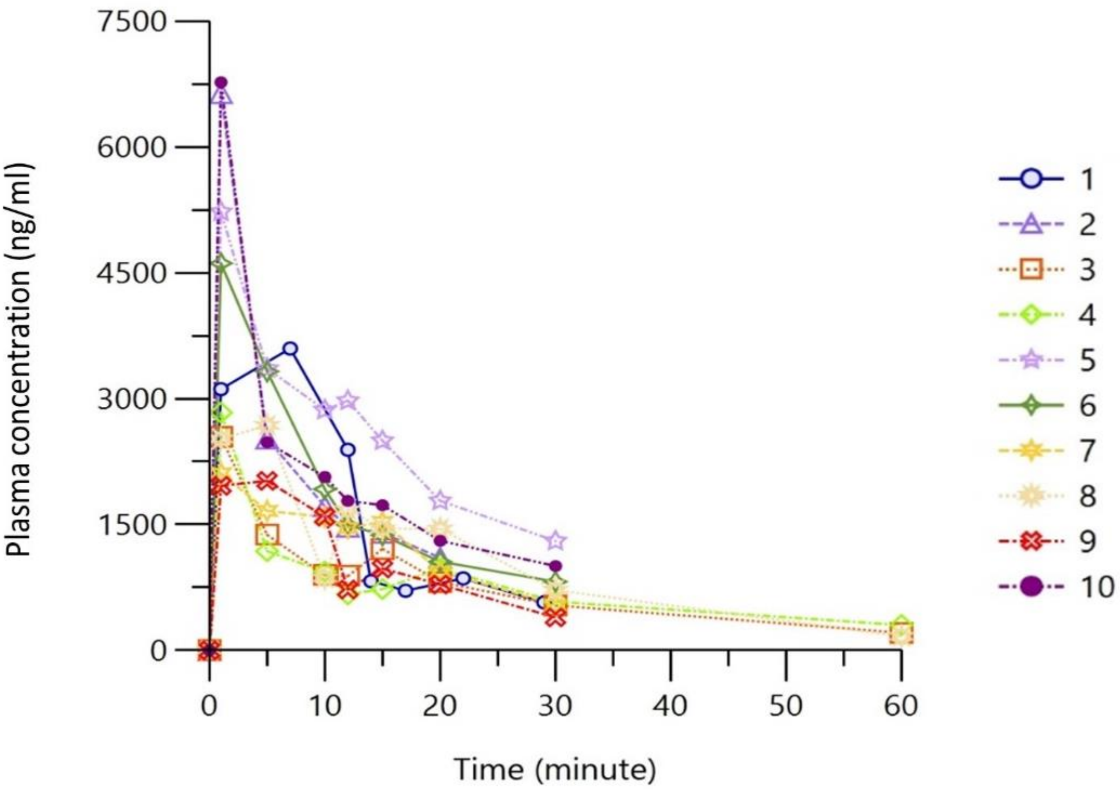

Figure S1 Spaghetti plot for cisatracurium plasma concentration-time profiles of ten critically ill patients
