# Supplementary material for: Pharmacokinetics and pharmacodynamics studies of a loading dose of cisatracurium in critically ill patients with respiratory failure
Source: BMC Anesthesiol. 2022 Jan 22;22:32. doi: 10.1186/s12871-022-01571-2 (PMC8783433; doi:10.1186/s12871-022-01571-2)
Supplement: Supplementary file 4 — Additional file 4: Table S4. Total plasma concentrations of laudanosine in 10 critically ill patients (ng/ml). [file 12871_2022_1571_MOESM4_ESM.pdf]

Pharmacokinetic study and therapeutic effect of a loading dose of cisatracurium in critically ill patients with respiratory failure

Table S4 Total plasma concentrations of laudanosine in 10 critically ill patients (ng/ml)

Subject	Time (minute)							
	**1**	**5**	**10**	**12**	**15**	**20**	**30**	**60**
**1**	385.7	363.4	346.3	252.1	160.1	197.5	164.4	-
**2**	904.8	685.3	515.4	438.4	478.9	353.6	-	-
**3**	703.6	346.3	161.8	266.0	246.4	201	177	147.0
**4**	283.4	195.9	84.5	82.2	115	112.7	107	119.0
**5**	937.3	758	443.6	461.6	510.5	359.9	351	-
**6**	320.8	307	130.6	149.2	178.5	90.4	173.2	-
**7**	587.1	767.9	760.6	638	760.4	556.8	374.9	-
**8**	585.1	771.5	246.9	549.3	474	614	347.6	159.1
**9**	341.1	662.8	487.5	186.7	295.5	262.8	205.9	-
**10**	522.5	232.3	204.5	226.1	240	188.5	227.4	-
**Mean ± SD**	557.1 ± 234.89	509 ± 239.38	338.2 ± 212.93	325.0 ± 184.8	345.9 ± 203.44	293.7 ± 177.56	236.5 ± 96.92	141.7 ± 20.57

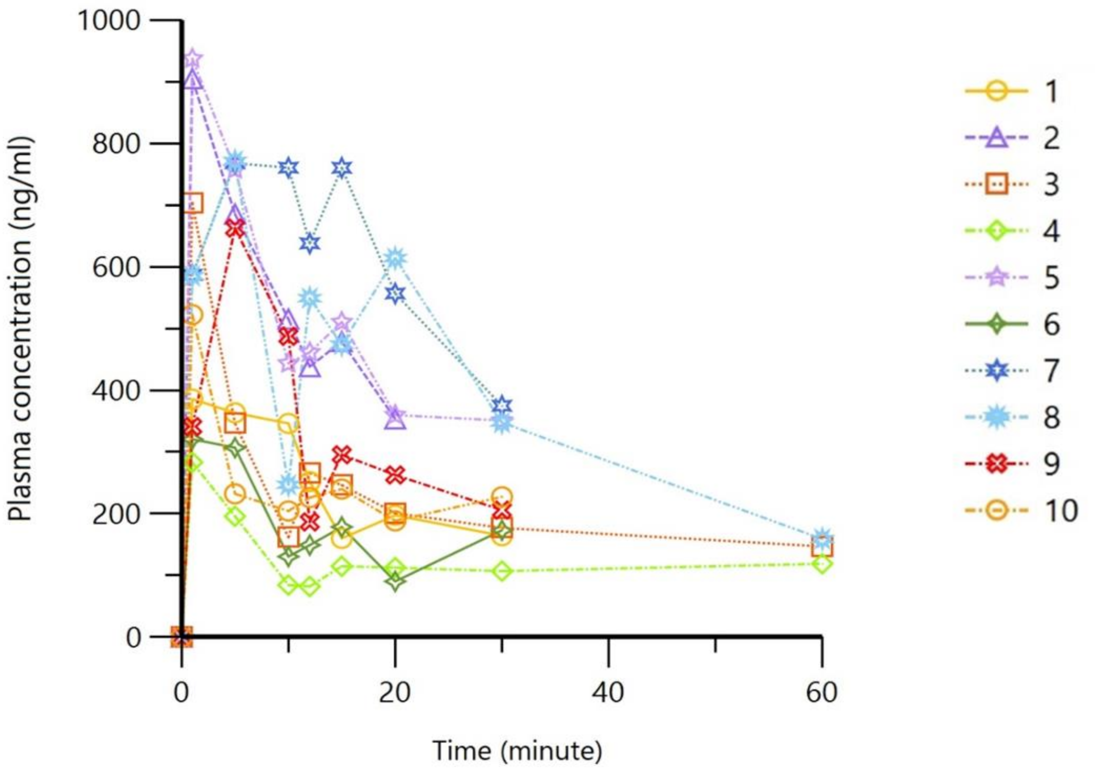

Figure S2. Spaghetti plot for laudanosine plasma concentration-time profiles of ten critically ill patients
