# Supplementary material for: Pharmacokinetics and pharmacodynamics studies of a loading dose of cisatracurium in critically ill patients with respiratory failure
Source: BMC Anesthesiol. 2022 Jan 22;22:32. doi: 10.1186/s12871-022-01571-2 (PMC8783433; doi:10.1186/s12871-022-01571-2)
Supplement: Supplementary file 5 — Additional file 5: Table S5 and S6. Correlation of patient characteristics and pharmacokinetic parameters. [file 12871_2022_1571_MOESM5_ESM.pdf]

Table S5. Correlation of patients's characteristics and pharmacokinetic parameters

		Age	Ht	BW	APACHE II	Scr	CrCl	BMI (kg/m2)	BT_M0	Alb (g/dL)	AST	ALT	ALP	GGT	TB	DB	24-hr fluid balance (ml)	PFratio	pH_M0	pCO2_M0	HCO3	Na	K	Mg	Ca
Cis_M1	Pearson Correlation	-.191	.564	.236	-.077	-.140	.032	-.060	.031	-.533	-.558	-.457	.565	-.385	.681*	.679*	-.472	.036	-.193	-.566	-.740*	-.152	-.286	-.185	.030
	Sig. (2-tailed)	.598	.090	.511	.833	.700	.930	.870	.932	.112	.093	.184	.089	.271	.030	.031	.168	.922	.593	.088	.014	.674	.424	.609	.935
	N	10	10	10	10	10	10	10	10	10	10	10	10	10	10	10	10	10	10	10	10	10	10	10	10
Cis_M5	Pearson Correlation	-.002	-.055	-.093	-.018	-.239	.109	-.081	-.226	-.158	.131	.339	.090	-.618	.019	.002	-.631	-.409	.090	.152	-.174	-.564	-.166	-.147	.093
	Sig. (2-tailed)	.995	.879	.799	.960	.506	.765	.825	.531	.663	.717	.338	.804	.057	.959	.996	.050	.240	.805	.674	.631	.090	.648	.685	.798
	N	10	10	10	10	10	10	10	10	10	10	10	10	10	10	10	10	10	10	10	10	10	10	10	10
Cis_M10	Pearson Correlation	.016	.005	-.047	-.067	-.403	.129	-.053	-.171	-.398	.042	-.050	.252	-.330	.122	.096	-.421	-.332	-.088	.119	-.209	-.370	-.221	.005	-.039
	Sig. (2-tailed)	.966	.989	.897	.854	.249	.723	.885	.636	.254	.909	.891	.483	.352	.737	.792	.225	.349	.810	.742	.563	.293	.540	.989	.914
	N	10	10	10	10	10	10	10	10	10	10	10	10	10	10	10	10	10	10	10	10	10	10	10	10
Cis_M12	Pearson Correlation	.169	-.031	.033	.219	-.222	.069	.081	-.137	-.105	-.450	-.275	-.082	-.305	-.184	-.176	-.092	-.238	-.412	.153	-.326	-.577	.070	.266	-.221
	Sig. (2-tailed)	.640	.932	.927	.543	.537	.850	.825	.706	.773	.192	.442	.822	.391	.612	.626	.800	.507	.237	.673	.357	.081	.848	.458	.539
	N	10	10	10	10	10	10	10	10	10	10	10	10	10	10	10	10	10	10	10	10	10	10	10	10
Cis_M15	Pearson Correlation	.238	-.088	-.065	.258	-.221	.071	-.013	-.074	-.048	-.439	-.393	-.056	-.182	-.207	-.204	.060	-.146	-.459	.098	-.361	-.571	.165	.284	-.249
	Sig. (2-tailed)	.508	.810	.859	.471	.540	.845	.971	.840	.895	.205	.262	.879	.615	.566	.571	.870	.688	.182	.788	.306	.085	.648	.426	.487
	N	10	10	10	10	10	10	10	10	10	10	10	10	10	10	10	10	10	10	10	10	10	10	10	10
Cis_M20	Pearson Correlation	.226	.197	.266	.230	.006	-.158	.252	-.328	-.091	-.621	-.151	-.080	-.491	-.171	-.153	-.057	-.113	-.215	.114	-.188	-.377	.063	.246	-.202
	Sig. (2-tailed)	.530	.584	.457	.523	.987	.662	.482	.355	.802	.055	.676	.826	.149	.637	.673	.876	.756	.550	.753	.603	.283	.862	.494	.576
	N	10	10	10	10	10	10	10	10	10	10	10	10	10	10	10	10	10	10	10	10	10	10	10	10
Cis_M30	Pearson Correlation	.093	.247	.132	.298	-.097	-.028	.025	-.438	-.351	-.619	-.380	.228	-.494	.087	.103	-.122	-.046	-.249	-.151	-.474	-.340	.082	.031	-.148
	Sig. (2-tailed)	.812	.521	.736	.436	.804	.943	.949	.239	.355	.076	.313	.555	.177	.825	.793	.755	.906	.519	.697	.198	.371	.834	.936	.704
	N	9	9	9	9	9	9	9	9	9	9	9	9	9	9	9	9	9	9	9	9	9	9	9	9
Vss_obs 1COM	Pearson Correlation	.112	.023	.318	-.014	.394	-.307	.419	-.003	.319	.048	.168	-.348	.435	-.303	-.282	.494	.200	.153	.263	.655*	.671*	.061	.196	.038
	Sig. (2-tailed)	.758	.949	.371	.970	.259	.389	.228	.993	.369	.895	.643	.325	.209	.394	.429	.147	.579	.674	.462	.040	.034	.866	.588	.918
	N	10	10	10	10	10	10	10	10	10	10	10	10	10	10	10	10	10	10	10	10	10	10	10	10
Vss_obs1COM(L/Kg)	Pearson Correlation	.087	-.413	-.187	.041	.133	.004	.018	.115	.416	.369	.138	-.450	.590	-.472	-.464	.618	.143	.081	.320	.576	.325	.267	.199	-.116
	Sig. (2-tailed)	.811	.235	.606	.910	.714	.991	.961	.751	.232	.295	.704	.192	.073	.168	.176	.057	.694	.824	.368	.081	.359	.456	.581	.749
	N	10	10	10	10	10	10	10	10	10	10	10	10	10	10	10	10	10	10	10	10	10	10	10	10
Ke 1COM	Pearson Correlation	-.300	.776**	.435	-.252	.066	-.103	.058	.112	-.475	-.412	-.202	.591	-.342	.873**	.872**	-.625	.091	.101	-.664*	-.526	.228	-.506	-.393	.237
	Sig. (2-tailed)	.400	.008	.209	.482	.856	.777	.874	.758	.166	.237	.575	.072	.333	.001	.001	.053	.803	.781	.036	.119	.527	.135	.262	.509
	N	10	10	10	10	10	10	10	10	10	10	10	10	10	10	10	10	10	10	10	10	10	10	10	10
Cl_obs 1COM	Pearson Correlation	-.080	.681*	.643*	-.116	.474	-.408	.405	.052	-.064	-.304	-.019	.179	.080	.429	.450	-.057	.247	.285	-.350	.189	.838**	-.376	-.272	.295
	Sig. (2-tailed)	.826	.030	.045	.749	.167	.241	.246	.888	.860	.394	.958	.621	.827	.216	.191	.875	.491	.425	.322	.601	.002	.284	.447	.409
	N	10	10	10	10	10	10	10	10	10	10	10	10	10	10	10	10	10	10	10	10	10	10	10	10
HL 1COM	Pearson Correlation	.089	-.721*	-.259	-.039	-.251	.243	.142	.208	.371	.335	.085	-.624	.563	-.719*	-.722*	.425	-.218	-.451	.748*	.366	-.290	.321	.685*	-.282
	Sig. (2-tailed)	.806	.019	.470	.914	.485	.499	.696	.563	.291	.345	.816	.054	.090	.019	.018	.221	.546	.191	.013	.299	.417	.365	.029	.429
	N	10	10	10	10	10	10	10	10	10	10	10	10	10	10	10	10	10	10	10	10	10	10	10	10
AUCall 1COM	Pearson Correlation	.191	-.347	-.065	.151	-.309	.139	.162	-.083	.022	-.118	-.117	-.294	-.027	-.463	-.464	.045	-.357	-.468	.517	-.022	-.534	.128	.462	-.231
	Sig. (2-tailed)	.597	.326	.858	.678	.385	.701	.654	.819	.951	.746	.748	.409	.941	.178	.177	.902	.312	.173	.126	.952	.112	.725	.179	.521
	N	10	10	10	10	10	10	10	10	10	10	10	10	10	10	10	10	10	10	10	10	10	10	10	10

**. Correlation is significant at the 0.01 level (2-tailed).

*. Correlation is significant at the 0.05 level (2-tailed).

Table S6. Correlation of patients's characteristics and pharmacokinetic parameters

		Shock	ARDS	AF	AKI	Anemia	Pneumonia	Septicemia	CVA	Delirium	UGIB	AP	Fen	Propofol	Mida	Acidosis	Met_acido	Res_alkalo
Cis_M1	Pearson Correlation	-.019	.328	.299	-.374	-.192	.340	.503	-.157	-.515	-.137	-.329	.247	.182	-.182	.155	.358	.485
	Sig. (2-tailed)	.958	.354	.401	.287	.596	.336	.138	.666	.128	.705	.353	.492	.615	.615	.669	.310	.155
	N	10	10	10	10	10	10	10	10	10	10	10	10	10	10	10	10	10
Cis_M5	Pearson Correlation	.173	.706*	.396	-.466	-.020	.514	-.161	.156	-.359	.486	-.313	.431	.618	-.618	-.203	.165	-.301
	Sig. (2-tailed)	.633	.022	.257	.175	.956	.128	.656	.667	.309	.154	.378	.214	.057	.057	.573	.648	.398
	N	10	10	10	10	10	10	10	10	10	10	10	10	10	10	10	10	10
Cis_M10	Pearson Correlation	.141	.609	.294	-.449	-.460	.435	.112	.058	-.073	.374	-.049	.418	.050	-.050	-.024	.048	-.280
	Sig. (2-tailed)	.697	.062	.410	.194	.180	.209	.759	.874	.840	.287	.892	.230	.890	.890	.948	.894	.433
	N	10	10	10	10	10	10	10	10	10	10	10	10	10	10	10	10	10
Cis_M12	Pearson Correlation	-.387	.472	-.189	-.138	-.116	.204	.174	-.218	-.232	-.290	.032	.260	-.036	.036	.052	.341	-.207
	Sig. (2-tailed)	.270	.168	.601	.704	.751	.572	.631	.546	.520	.416	.930	.469	.921	.921	.886	.335	.566
	N	10	10	10	10	10	10	10	10	10	10	10	10	10	10	10	10	10
Cis_M15	Pearson Correlation	-.326	.394	-.308	.037	-.233	.155	.255	-.189	-.108	-.437	.115	.102	-.211	.211	.106	.259	-.146
	Sig. (2-tailed)	.359	.260	.387	.919	.517	.668	.477	.601	.766	.207	.752	.779	.559	.559	.771	.470	.687
	N	10	10	10	10	10	10	10	10	10	10	10	10	10	10	10	10	10
Cis_M20	Pearson Correlation	-.473	.351	-.209	-.160	.115	.054	.021	-.308	-.405	-.275	-.184	.322	.026	-.026	-.141	.356	-.184
	Sig. (2-tailed)	.167	.320	.562	.658	.752	.882	.955	.386	.245	.441	.610	.364	.944	.944	.697	.312	.611
	N	10	10	10	10	10	10	10	10	10	10	10	10	10	10	10	10	10
Cis_M30	Pearson Correlation	-.225	.322	-.194	-.332	-.036	.296	.111	-.226	-.500	-.194	-.231	.241	-.042	.042	-.231	.431	.053
	Sig. (2-tailed)	.560	.399	.617	.382	.927	.439	.776	.558	.170	.617	.550	.532	.914	.914	.550	.247	.892
	N	9	9	9	9	9	9	9	9	9	9	9	9	9	9	9	9	9
Vss_obs 1COM	Pearson Correlation	-.277	-.671*	-.208	.225	.221	-.499	-.284	-.199	.289	-.044	.171	-.219	-.377	.377	-.047	-.215	-.114
	Sig. (2-tailed)	.439	.034	.564	.532	.540	.142	.427	.581	.418	.904	.637	.543	.282	.282	.897	.552	.754
	N	10	10	10	10	10	10	10	10	10	10	10	10	10	10	10	10	10
Vss_obs1COM(L/Kg)	Pearson Correlation	-.050	-.522	-.410	.498	.141	-.498	-.239	.087	.585	-.149	.431	-.348	-.416	.416	.025	-.348	-.199
	Sig. (2-tailed)	.891	.121	.239	.143	.697	.143	.506	.811	.076	.681	.213	.324	.231	.231	.946	.324	.582
	N	10	10	10	10	10	10	10	10	10	10	10	10	10	10	10	10	10
Ke 1COM	Pearson Correlation	.091	.091	.591	-.455	-.020	.273	.377	-.091	-.545	.091	-.515	.212	.408	-.408	.136	.212	.636*
	Sig. (2-tailed)	.803	.803	.072	.187	.957	.446	.283	.803	.103	.803	.128	.556	.242	.242	.707	.556	.048
	N	10	10	10	10	10	10	10	10	10	10	10	10	10	10	10	10	10
Cl_obs 1COM	Pearson Correlation	-.115	-.619	.313	-.213	.201	-.139	-.047	-.203	-.239	.040	-.369	-.119	.012	-.012	.006	-.051	.481
	Sig. (2-tailed)	.752	.056	.378	.555	.577	.701	.898	.573	.506	.913	.293	.743	.974	.974	.987	.889	.160
	N	10	10	10	10	10	10	10	10	10	10	10	10	10	10	10	10	10
HL 1COM	Pearson Correlation	-.401	.087	-.424	.385	-.149	-.323	.026	-.148	.634*	-.138	.794**	.007	-.418	.418	.275	-.051	-.672*
	Sig. (2-tailed)	.250	.810	.223	.272	.682	.362	.944	.683	.049	.705	.006	.985	.230	.230	.442	.888	.033
	N	10	10	10	10	10	10	10	10	10	10	10	10	10	10	10	10	10
AUCall 1COM	Pearson Correlation	-.404	.438	-.221	-.091	-.251	.136	.059	-.215	.094	-.090	.337	.254	-.213	.213	.099	.212	-.543
	Sig. (2-tailed)	.248	.206	.540	.803	.484	.707	.872	.552	.796	.806	.340	.479	.554	.554	.785	.557	.105
	N	10	10	10	10	10	10	10	10	10	10	10	10	10	10	10	10	10

**. Correlation is significant at the 0.01 level (2-tailed).

*. Correlation is significant at the 0.05 level (2-tailed).
